# How to choose kinematic or mechanical alignment individually according to preoperative characteristics of patients?

**DOI:** 10.1186/s12891-020-03472-2

**Published:** 2020-07-07

**Authors:** Chong Luan, Dong-Tan Xu, Ning-Jie Chen, Fei-Fei Wang, Kang-Song Tian, Chao Wei, Xian-Bin Wang

**Affiliations:** Department of Orthopaedic Surgery, ZiBo central hospital, 54 Gong Qingtuan West Road, Zhangdian, Zibo, 255036 Shandong People’s Republic of China

**Keywords:** Total knee arthroplasty, Kinematic alignment, Mechanical alignment, Knee society score

## Abstract

**Background:**

Making decisions in alignment techniques in total knee arthroplasty (TKA) remains controversial. This study aims to identify the potential patients who were suitable for the kinematic (KA) or mechanical alignment (MA).

**Methods:**

We reviewed 296 consecutive patients (296 TKAs, including 114 KA-TKAs and 182 MA-TKAs) who underwent unilateral TKA using a computer-assisted navigation from 2016 to 2018 in our prospectively maintained database. The minimum followup was 1 year. Clinical outcomes including the range of motion (ROM) and knee society score (KSS) were compared between KA-TKAs and MA-TKAs. Multiple regression models were used to evaluate the relationship between alignment techniques and KSS at the 1-year followup. Interaction and stratified analyses were conducted according to gender, age, body mass index (BMI), preoperative hip-knee-ankle (HKA) angle, ROM and KSS.

**Results:**

ROM and KSS at the 1-year followup didn’t differ between MA-TKAs and KA-TKAs (all *p* > 0.05). Alignment techniques did not associate with postoperative ROM (Adjusted β = 0.4, 95% confidence interval [CI]: − 0.3, 1.6; *p* = 0.752) or 1-year KSS (Adjusted β = 2.2, 95%CI: − 0.7, 5.6; *p* = 0.107). Patients with a BMI more than 30 kg/m^2 achieved better 1-year KSS when using MA than KA (p for interaction< 0.05). Additionally, patients with preoperative HKA angle more than 10 degrees varus benefited more from KA than MA (p for interaction< 0.05).

**Conclusions:**

Patients with severe varus deformity may be suitable for the KA technique, whereas MA should be used in obese patients.

## Background

Total knee arthroplasty (TKA) is considered to be one of the most successful orthopedic surgery for pain relief and functional recovery in patients with knee arthritis. However, surgeons’ perceptions of the success of the operationon are discordant with those of patients. Recently, many surgeons pay more attention on the patient-reported outcome measures (PROMs) to evaluate the results of the procedure [1]. According to the PROMs, it has reported that about 20% of patients with TKA were dissatisfied with the clinical outcomes of TKA [2, 3].

One possible explanation for these dissatisfied patients is that contemporary TKA techniques fail to restore the nature knee kinematics [4]. Thus, there is an increasing debate regarding optimal alignment in TKA. Mechanical alignment (MA), the classical method proposed by Insall et al. [5], aims to create a neutral hip-knee-ankle (HKA) axis. Kinematic alignment (KA) in TKA is an alternative technique to MA, which attempts to maintain the natural kinematic axis and ligament balance of the individual knee. Some studies indicated that the KA technique achieved a greater range of motion (ROM) and a higher rate of postoperative satisfaction compared with MA in TKA [6, 7]. However, several researches suggested similar results with these two alignment techniques [8]. Hence, we speculate that the KA or MA alignment technique may not be suitable for every case, which means surgeons should choose KA or MA individually.

To our best knowledge, there has been a lack of study on indications for the alignment parameter. Therefore, we conduct this study to identify the potential patients who were suitable for KA or MA technique in TKA. Additionally, we aimed to compare clinical outcomes in KA-TKA or MA-TKA using a multiple regression analysis with an adjustment for potential confounders.

## Methods

### Patients

After the Institutional Review Board approval, we reviewed 367 consecutive patients who underwent unilateral TKA using a portable navigation system from 2016 to 2018 in our prospectively maintained institutional navigation TKA database. The indication for TKA was according to the patient’s symptoms, X-ray, and the surgeon’s discretion. All procedures were performed by three senior surgeons with extensive experience in navigation assisted TKA, including one surgeon routinely using KA techniques and two routinely using MA techniques. We excluded patients with post-traumatic, septic or inflammatory arthritis of the knee, BMI > 40 kg/m^2, patients with valgus knee, patients with contralateral TKA, or ipsilateral THA, those without a minimum followup of 1 year. After the aforementioned exclusion, 296 patients (296 TKAs, including 114 KA-TKAs and 182 MA-TKAs) were included in the final analysis.

### Surgical techniques

All procedures were performed using the PS Vanguard TKA (Zimmer-Biomet, Warsaw, Indiana, USA). The medial parapatellar approach was conducted after placing tracker pins. The I-Assist navigation system (Zimmer-Biomet, Warsaw, Indiana, USA) was used to achieve coronal plane alignment according to surgeons’ preference. In the MA-TKA, a neutral HKA axis with perpendicular components to the femoral and tibial mechanical axes was created. Femoral external rotation was set at 3° to the femoral posterior condyles. With regard to the KA-TKA, a modified KA protocol described by Hutt et al. [9] was conducted in our institution. The bone cut was modified from patient anatomy, which was conducted as planned using the portable navigation system. The accuracy of bone resection was evaluated by caliper measurements. The HKA angles were limited for the KA-TKA from 6° varus to 3° valgus. The bone cut of the posterior condyle was equal to the component thickness and matched with individual native femoral rotation. The tibial rotation was set relative to the femoral trial component with the knee in extension. We did not routinely resurface the patellar in our institution. The postoperative care, including antibiotic administration, anticoagulation, and physiotherapy, was based on an institutional protocol in all patients.

Radiographic evaluations using standing full-leg radiographs were performed and evaluated by two trained orthopedic fellows preoperatively and at the 1-year followup to determine the HKA angle. The inter-observer reliability were calculated by the interclass correlation coefficient (ICC). The Inter-observer agreements were 0.93, and the mean of the two observers’measurements was used for the following analysis. Other medical records were reviewed manually to extract pertinent variables.

The primary outcome was Knee Society Score (KSS) 2011 [10], including symptoms (0–25), satisfaction (0–40), expectation (3–15), and functional activities (0–100) at the 1-year followup. Secondary outcomes included the range of motion (ROM) and the HKA angle.

### Statistical analysis

Statistical analysis was performed by using the statistical software packages R (http://www.R-project.org). The student t-test was used to compare continuous variables of the normal distribution, including BMI and age. The ROM, HKA angel, KSS score were not normal distribution, and the Mann-Whitney test was conducted. Fisher’s exact test for categorical variables. Multiple regression analyses were conducted to evaluate the independent relationship between alignment methods and outcomes. The interaction and stratified analyses were used to identify patients who were suitable for KA or MA technique in TKA. A two-piecewise linear regression model was conducted, and adjusted smoothing spline plots were created to graphically depict the associations between continuous variables and KSS scores at 1-year followup. A *p*-value of less than 0.05 was considered significant.

## Results

The patient characteristics were presented in Table 1. There was no significant difference in age, BMI, gender, or ASA score among patients with KA-TKA or MA-TKA. Patients in the KA-TKA group had higher degrees of varus deformity compared with those in the MA-TKA group (9.6 ± 8.1 varus vs. 7.2 ± 5.3 varus, *p* = 0.046). Preoperative ROM and KSS scores were comparable between groups.

**Table 1:** Patient demographics

	KA-TKA Group (*n* = 114)	MA-TKA Group (*n* = 182)	*P*-value
Age (year)(mean ± SD)	65.65 ± 13.4	64.92 ± 14.73	0.419
BMI (kg/m^2^) (mean ± SD)	28.18 ± 2.61	28.06 ± 3.36	0.126
Female (n, %)	86 (75.4%)	129 (70.9%)	0.069
ASA score ≥ 3 (n, %)	13 (11.4%)	23 (12.6%)	0.527
Preoperative (mean ± SD)			
ROM (°)	109 ± 19.1	112 ± 20.7	0.963
HKA angle (°)	9.6 ± 8.1 varus	7.2 ± 5.3 varus	0.046*
KSS symptom (25)	9.1 ± 3.8	8.9 ± 4.2	0.857
KSS satisfaction (40)	15.6 ± 7.2	14.9 ± 5.2	0.461
KSS expectation (15)	12.8 ± 1.7	13.1 ± 2.2	0.581
KSS functional activities (100)	38.3 ± 15.7	41.6 ± 14.3	0.265
Total KSS score (180)	73.2 ± 30.4	79.5 ± 28.6	0.624

Clinical outcomes at the 1-year followup didn’t differ between groups (Table 2). The postoperative ROM of KA and MA was (125.6 ± 19.1) degrees and (124.9 ± 17.7) degrees, respectively. The total KSS of KA and MA was (126.8 ± 16.4) degrees and (124.3 ± 14.3) degrees, respectively. After adjusting for confounding variables in Table 1, the clinical outcomes showed no significant difference in ROM (Adjusted β = 0.4, 95%CI: − 0.3, 1.6; *p* = 0.752) or total KSS (Adjusted β = 2.2, 95%CI: − 0.7, 5.6; *p* = 0.107) between groups. Due to differing targets for alignment, the postoperative HKA angle of KA-TKA and MA-TKA was significantly different (1.2 ± 2.5 vs. 0.3 ± 1.9, *p* = 0.027). One case in the MA group developed periprosthetic joint infection after TKA and then underwent debridement, antibiotics, and implant retention. There was no other reoperation in both groups.

**Table 2:** Clinical outcomes among patients with KA or MA at the 1-year followup

Parameters (mean ± SD)	KA-TKA Group (*n* = 114)	MA-TKA Group (*n* = 182)	#Adjusted β (95% CI)	*P*-value
ROM (°)	125.6 ± 19.1	124.9 ± 17.7	0.4 (−0.3, 1.6)	0.752
HKA angle (°)	1.2 ± 2.5 varus	0.3 ± 1.9 varus	1.5 (0.3,1.7)	0.027*
KSS symptom (25)	18.1 ± 4.5	18.7 ± 4.7	−0.6 (−1.3, 0.5)	0.916
KSS satisfaction (40)	25.6 ± 6.1	25.9 ± 6.7	−0.2 (− 0.6, 1.2)	0.639
KSS expectation (15)	10.2 ± 2.6	9.9 ± 1.8	0.5 (−0.8, 2.1)	0.092
KSS functional activities (100)	66.4 ± 6.9	65.7 ± 7.1	1.1 (−0.9, 2.7)	0.374
Total KSS score (180)	126.8 ± 16.4	124.3 ± 14.3	2.2 (−0.7, 5.6)	0.107

#MA-TKA was considered as the reference

**Fig. 1:**
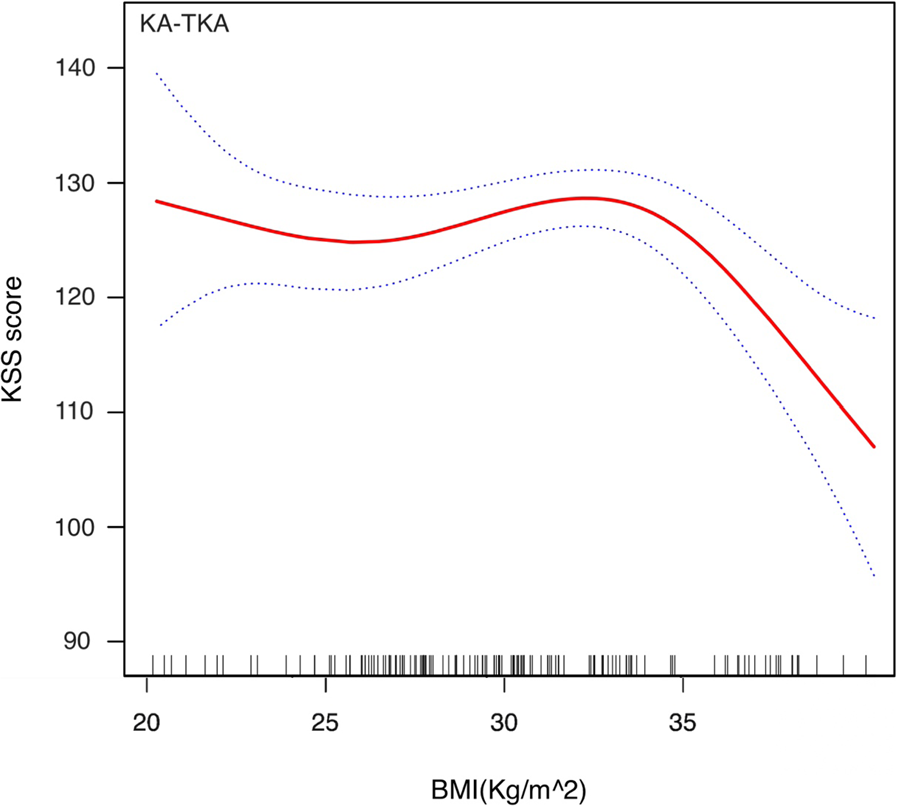
Adjusted smoothing spline between BMI and 1-year KSS in the KA-TKA group. The stippled lines indicate the 95% CIs. Short vertical lines on the x-axis represent individual case in the study

**Fig. 2:**
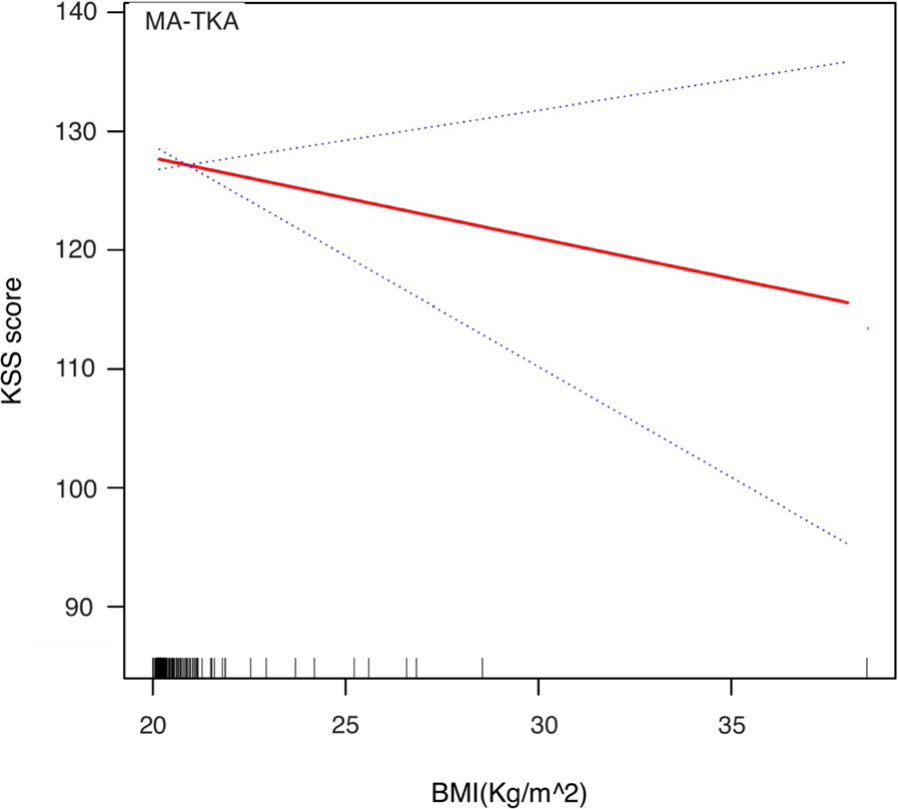
Adjusted smoothing spline between BMI and 1-year KSS in the MA-TKA group. The stippled lines indicate the 95% CIs. Short vertical lines on the x-axis represent individual case in the study

**Fig. 3:**
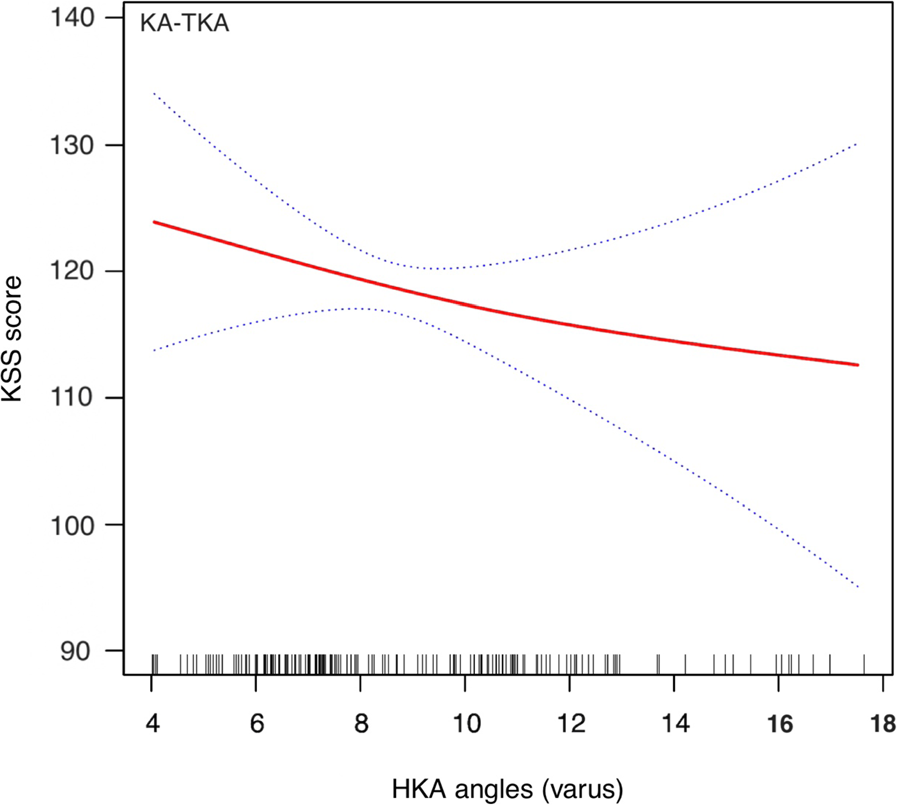
Adjusted smoothing spline between preoperative HKA angle (varus deformity) and 1-year KSS in the KA-TKA group. The stippled lines indicate the 95% CIs. Short vertical lines on the x-axis represent individual case in the study

**Fig. 4:**
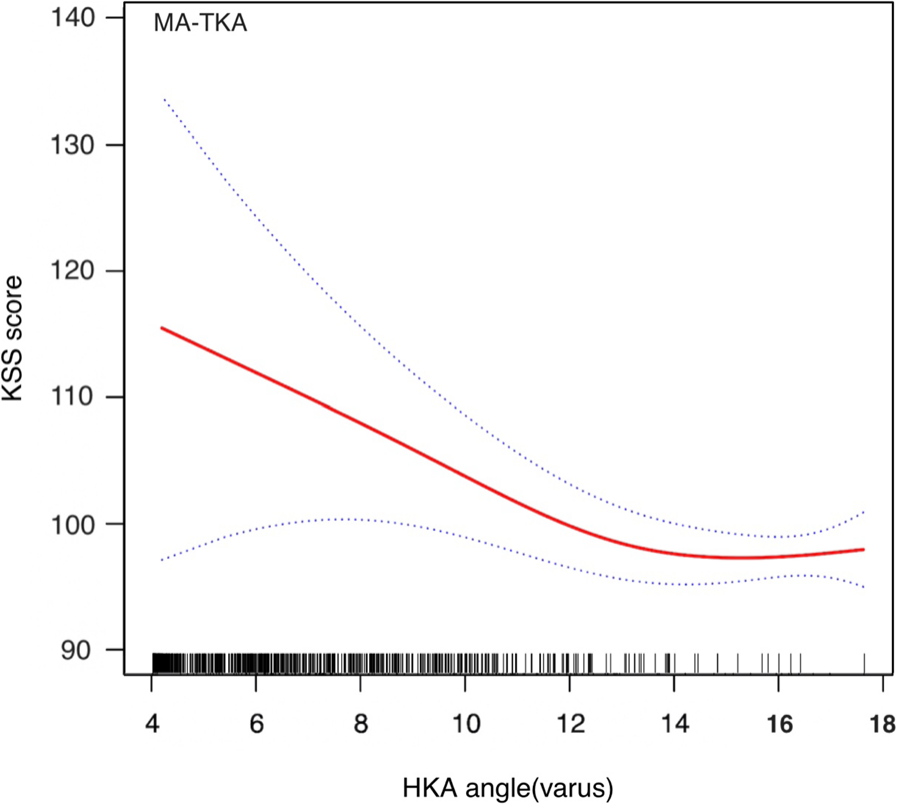
Adjusted smoothing spline between HKA preoperative angle (varus deformity) and 1-year KSS in the MA-TKA group. The stippled lines indicate the 95% CIs. Short vertical lines on the x-axis represent individual case in the study

The stratified and Interaction analyses suggested that the association between alignment techniques (MA as reference) and total KSS score was modified by obesity and preoperative HKA angle (Table 3). Patients with BMI ≥ 30 kg/m^2^ had a lower β between the KA-TKA and KSS than those with BMI < 30 kg/m (β: − 1.66 vs. 1.48, p for interaction = 0.028). Additionally, the β between the KA-TKA and KSS was higher in patients with preoperative HKA ≥ 10 degrees varus than those with HKA < 10 degrees varus (β: 2.44 vs. 0.52, p for interaction = 0.013). The adjusted smoothing splines were revealed the relationship between BMI (Figs. 1 and 2) or preoperative HKA angle (Figs. 3 and 4) and total KSS stratified by alignment techniques. Figure 1 showed a nonlinear relationship between BMI and KSS in patients with KA-TKA. When BMI was higher than the turning point at a BMI of 32.5 kg/m^2, the KSS decreased significantly with the increase in BMI (Fig. 1). Additionally, there were nonlinear relationships between preoperative HKA angle and 1-year KSS. (Fig. 2) The two-piecewise linear regression model didn’t find the turning point.

**Table 3:** Interaction and stratified analyses between alignment techniques (MA as reference) and KSS score

Subgroup	β, 95% CI	*P*-value	*P*-value for interaction
*Sex*			0.684
Male	1.01 (−1.06, 2.08)	0.793	
Female	1.48 (−4.89, 6.92)	0.813	
*Age, years*			0.076
< 65	1.53 (−2.40, 5.45)	0.793	
≥ 65	1.07 (−1.30, 2.76)	0.452	
BMI, kg/m^2			0.028*
< 30	1.48 (−1.81, 5.17)	0.274	
≥ 30	−1.66 (−4.08, −0.46)	0.039*	
*Preoperative HKA angle*			0.013*
< 10° varus	0.52 (−0.99, 0.95)	0.983	
≥ 10° varus	2.44 (0.53, 4.63)	0.042*	
*Preoperative ROM*			0.668
< 90°	0.82 (−1.74, 2.59)	0.702	
≥ 90°	0.42 (−2.48, 3.31)	0.78	
*Preoperative total KSS*			0.583
Tertile low	−0.84 (−2.04, −0.15)	0.328	
Tertile middle	1.04 (−0.12, 3.21)	0.565	
Tertile high	0.21 (−2.13, 2.55)	0.859

## Discussion

Recently, there has been a rising debate on MA or KA techniques. The data in the literature was inconsistent, which makes surgeons confused about making decisions. This may be explained that we should choose alignment techniques individually. The most important finding of the present study was that patients with preoperative varus deformity more than 10 degrees were more suitable for KA-TKA, while patients with BMI more than 30 kg/m^2 would benefit more from MA-TKA.

There have been numerous studies on the comparison between the MA-TKA and the KA-TKA. Several studies have suggested a substantial portion of the normal population didn’t have a neutral mechanical alignment. Bellemans et al. reported 32% of men and 17% of women had varus knees with a natural mechanical axis of 3° varus or more [11]. Nam et al. suggested that only 31% of knees had both a neutral mechanical alignment and the absence of joint line obliquity [12]. Thus, several scholars hold the view that KA-TKA may restore normal knee kinematics. Faschingbauer et al. indicated KA-TKA could achieve similar kinematics of the patellofemoral joint relative to the normal state [13]. Blakeney et al. suggested KA-TKA reproduced more closely normal gait of healthy controls compared to MA-TKA due to the restoration of the individual’s knee kinematics and ligament tension in KA-TKA [14]. Ishikawa et al. revealed more significant femoral rollback and more external rotation of the femoral component in KA-TKA than Ma-KTA by using a musculoskeletal computer simulation [15].

Although the studies above provided evidence in favor of KA-TKA, the present study found the clinical outcomes were comparable between the two groups, which were similar to data in the recent literature. Luo et al. conducted a meta-analysis including nine randomized controlled trials with 1170 KA-TKAs and 1171 MA-TKAs. This meta-analysis suggested the KSS, knee injury, and osteoarthritis outcome score (KOOS), EuroQoL 5-dimension questionnaire (ED-5D), ROM, and complications were similar for KA-TKA and MA-TKA. In a recent study by McEwen et al. [16], they prospectively enrolled 41 patients who were scheduled to undergo simultaneous TKAs. They randomized one side using MA and the other side using KA. With a minimum of 2-year followup, although more patients preferred their KA knees, they suggested no difference in ROM or functional scores between groups. Additionally, there is a lack of data on comparisons the long-term clinical outcomes. Ishikawa et al. [15] suggested KA-TKA increased patellofemoral and tibiofemoral contact stresses by using finite element analysis, which may impair long-term outcomes. Berend et al. reviewed 3152 TKAs for osteoarthritis with a mean 5-year followup and indicated that varus tibial component alignment more than 3.0 degrees had a 17-fold risk of subsequent tibial component aseptic loosening [17]. Another study with the mean followup of 7.6 years found failure was least likely to occur in patients with a neutral alignment of both the tibial and the femoral component [18].

Several studies have reported the survival rate of TKA was lower in obese patients than nonobese patients [19–21]. However, the interaction between obesity and alignment on the survival of TKA remains unknown. The present study revealed that obese patients might be more suitable for MA-TKA than KA-TKA. Interestingly, our results were similar to a previous study by Berend et al. [17]. They found BMI alone was not associated with failure, but BMI more than 33.7 kg/m^2 combined with varus tibial component more than 3 degrees had a 168-fold risk of subsequent failure. The possible reason may be that overloading of the knee occurs in patients with high BMI and varus axis, resulting in more significant impact loading across the tibial component, therefore, caused patients’ discomfort and might increase component loosening and lower implant survival rate. Additionally, we found patients with preoperative HKA angle more than 10 degrees varus may benefit more from KA-TKA than MA-TKA. The reason remains unclear. It may be explained that patients with severe deformity frequently possessed critical contracture of knees. Thus, these patients need more soft tissue releases that may impair patients’ satisfaction.

Several limitations should be noted. First, this is a single-institution study, and thus its findings need external validation. Additionally, long-term outcomes were unknown. Second, only a single implant manufacturer was used in the present study, which may limit the generalizability of the findings. Third, the sample size may be inadequate for conducting some statistical analyses, and the possibility of a type-II error exists. Fourth, the angles of femoral and tibial cut were not considered in the present study. Lastly, there is potential variability in patient selections among surgeons. Given the lack of evidence and literature on who to undergo a MA-TKA or KA-TKA, there is no standardized protocol, and thus surgeon’s preference may be a factor, which may introduce bias.

## Conclusions

In conclusion, KA-TKA may not be suitable for obese patients, whereas patients with severe varus deformity may benefit more from KA-TKA. These findings need to be borne in mind when deciding which alignment techniques should be used in TKA. Further studies with the long-term followup are required to validate our results.

## Data Availability

Data are available on request from the authors.
